# Chronic Exposure to Cadmium and Antioxidants Does Not Affect the Dynamics of Expanded CAG•CTG Trinucleotide Repeats in a Mouse Cell Culture System of Unstable DNA

**DOI:** 10.3389/fncel.2020.606331

**Published:** 2021-02-02

**Authors:** Mário Gomes-Pereira, Darren G. Monckton

**Affiliations:** ^1^Sorbonne Université, Inserm, Centre de Recherche en Myologie, Paris, France; ^2^Institute of Molecular, Cell and Systems Biology, College of Medical, Veterinary and Life Sciences, University of Glasgow, Glasgow, United Kingdom

**Keywords:** trinucleotide repeat, unstable DNA, mismatch repair, oxidative stress, cadmium, antioxidants, cell culture model

## Abstract

More than 30 human disorders are caused by the expansion of simple sequence DNA repeats, among which triplet repeats remain the most frequent. Most trinucleotide repeat expansion disorders affect primarily the nervous system, through mechanisms of neurodysfunction and/or neurodegeneration. While trinucleotide repeat tracts are short and stably transmitted in unaffected individuals, disease-associated expansions are highly dynamic in the germline and in somatic cells, with a tendency toward further expansion. Since longer repeats are associated with increasing disease severity and earlier onset of symptoms, intergenerational repeat size gains account for the phenomenon of anticipation. In turn, higher levels of age-dependent somatic expansion have been linked with increased disease severity and earlier age of onset, implicating somatic instability in the onset and progression of disease symptoms. Hence, tackling the root cause of symptoms through the control of repeat dynamics may provide therapeutic modulation of clinical manifestations. DNA repair pathways have been firmly implicated in the molecular mechanism of repeat length mutation. The demonstration that repeat expansion depends on functional DNA mismatch repair (MMR) proteins, points to MMR as a potential therapeutic target. Similarly, a role of DNA base excision repair (BER) in repeat expansion has also been suggested, particularly during the removal of oxidative lesions. Using a well-characterized mouse cell model system of an unstable CAG•CTG trinucleotide repeat, we tested if expanded repeat tracts can be stabilized by small molecules with reported roles in both pathways: cadmium (an inhibitor of MMR activity) and a variety of antioxidants (capable of neutralizing oxidative species). We found that chronic exposure to sublethal doses of cadmium and antioxidants did not result in significant reduction of the rate of trinucleotide repeat expansion. Surprisingly, manganese yielded a significant stabilization of the triplet repeat tract. We conclude that treatment with cadmium and antioxidants, at doses that do not interfere with cell survival and cell culture dynamics, is not sufficient to modify trinucleotide repeat dynamics in cell culture.

## Introduction

The expansion of simple sequence DNA repeats has been implicated in human disease (Hannan, 2018). Trinucleotide repeat expansions, in particular, can cause severe neurological and/or neuromuscular disorders, including Huntington disease (HD), various spinocerebellar ataxias (SCA), fragile X syndrome (FRAXA) and myotonic dystrophy type 1 (DM1) (Paulson, 2018). In non-affected individuals, repeat tracts are short and genetically stable; in contrast affected individuals carry long and unstable expansions. Expansion mutations generally result in complex, multi-systemic phenotypes that are modulated by the number of tandem repeats: generally, longer triplet repeat alleles are associated with an earlier age at disease onset and more severe disease symptoms (Usdin et al., 2015). The majority of trinucleotide repeat diseases are caused by the expansion of CAG•CTG repeats, which can either encode a toxic polyglutamine tract in various proteins (Stoyas and La Spada, 2018), or be transcribed into a non-coding CUG RNA that interferes with the activity of key RNA-binding proteins and RNA processing (Sicot and Gomes-Pereira, 2013; Swinnen et al., 2020). Strikingly, all these conditions exhibit some element of neurological dysfunction, demonstrating the vulnerability of the central nervous system to triplet repeat expansion mutations (Orr and Zoghbi, 2007).

Once within the disease-associated size range, trinucleotide repeat alleles are highly unstable and liable to further expansion in germline transmissions and in somatic tissues (Usdin et al., 2015). Intergenerational instability frequently results in the inheritance of a longer repeat allele by the offspring, relative to the transmitting parent, hence explaining the phenomenon of anticipation. Anticipation presents as the aggravation of the disease symptoms and earlier age of onset in subsequent generations, and it is a typical feature of most trinucleotide repeat expansion disorders. Repeat instability in somatic tissues is also expansion-biased and age-dependent. Notably, the largest somatic expansions are often observed in the most severely affected tissues, including the striatum in HD (Kennedy and Shelbourne, 2000; Kennedy et al., 2003) and the skeletal muscle in DM1 (Aslanidis et al., 1992; Ashizawa et al., 1993; Thornton et al., 1994; Monckton et al., 1995). Age of onset and disease progression are most likely governed by the number of inherited repeats and the rate of repeat expansion in the soma. In support of this view, higher individual-specific repeat expansion rates have been directly linked with increased disease severity and earlier age of onset in HD and DM1 (Swami et al., 2009; Morales et al., 2012; Ciosi et al., 2019; Flower et al., 2019). Somatic repeat expansion thus likely contributes toward the tissue specificity and progressive nature of these disorders and presents as a potential therapeutic target (Gomes-Pereira and Monckton, 2006; Castel et al., 2010).

Given the relationship between repeat length and the disease severity, deciphering the mechanisms of trinucleotide repeat size mutation is relevant for understanding disease progression, and it may provide an entry point for future therapies. Disease-associated repeats form unusual secondary non-B DNA structures (Khristich and Mirkin, 2020), which may serve as substrates for DNA-binding proteins that mediate repeat size gains by error-prone processing. Several DNA repair pathways, which normally protect the genome against damage by normal metabolic activities and environmental factors, have been implicated in the mechanisms of repeat instability (Usdin et al., 2015). Among those, DNA mismatch repair (MMR) proteins are the strongest driver of repeat expansions (Schmidt and Pearson, 2016; Jones et al., 2017). Compelling evidence demonstrates that genetic inactivation of MMR genes suppresses repeat instability in mouse models of unstable CAG•CTG DNA (Manley et al., 1999; Van Den Broek et al., 2002; Gomes-Pereira et al., 2004b; Savouret et al., 2004; Foiry et al., 2006; Dragileva et al., 2009; Pinto et al., 2013), while natural polymorphisms in MMR genes dictate differences in repeat instability between mouse strains (Pinto et al., 2013; Tomé et al., 2013). Similarly, polymorphisms in human DNA mismatch repair genes are associated with individual-specific differences in CTG•CAG repeat instability (Morales et al., 2016; Ciosi et al., 2019; Flower et al., 2019) and in disease course in HD, DM1 and SCA patients (Lee et al., 2015, 2017, 2019; Bettencourt et al., 2016; Moss et al., 2017) The first demonstration that inhibition of repeat expansion could have therapeutic benefits was provided by the genetic inactivation of MMR in HD mice, which not only prevented accumulation of somatic mosaicism but also delayed brain pathology (Wheeler et al., 2003). Full MMR inactivation would however be unacceptable, given the expected increase in cancer predisposition. Interestingly, different levels of somatic instability between tissues have been correlated with physiological differences in the expression of MMR proteins (Mason et al., 2014). Therefore, it is conceivable that the controlled modulation of the relative expression and/or activity of the MMR complexes by non-toxic compounds would be sufficient to decrease repeat expansion rates and reduce mean repeat numbers in somatic cells. In this regard, partial inhibition of MMR by environmentally relevant concentrations of cadmium increased the frequency of mutations of a mononucleotide microsatellite, as well as frameshifts and base substitutions (Jin et al., 2003). The consequences of cadmium exposure on the dynamics of expanded trinucleotide DNA sequences remain to be determined.

An additional layer of complexity is added by the suggested role of oxidative stress and base excision repair (BER) in the generation of repeat expansions, following the observation that treatment of mouse and cell models of HD and FRAXA with oxidants appeared to increase repeat expansion (Kovtun et al., 2000, 2007; Entezam et al., 2010; Jonson et al., 2013). Conversely, the neutralization of oxygen species by a synthetic radical scavenger designed to target mitochondria appeared to reduce somatic instability (Budworth et al., 2015) and improve HD-associated pathology in a mouse model of the disease (Xun et al., 2012; Polyzos et al., 2018). However, the toxicology of this compound has not been fully investigated and its therapeutic application is still uncertain. If oxidative stress creates substrates for DNA mis-repair and repeat expansion, we hypothesize that other canonical antioxidants would produce similar protective effects.

We have previously generated a cell culture model that reproduces time-dependent, expansion-biased tissue-specific somatic mosaicism (Gomes-Pereira et al., 2001; Gomes-Pereira and Monckton, 2004) derived from a transgenic mouse model of unstable CAG•CTG repeats (Fortune et al., 2000). In this cell model, repeat expansion rates are not accounted for by different cell proliferation rates (Gomes-Pereira et al., 2001), and repeat size gains continue to occur even in the absence of cell division and genome duplication (Gomes-Pereira et al., 2014). Here, we have first investigated whether naturally occurring changes in the expression levels of MMR correlate with the degree of somatic mosaicism in homogenous cell cultures. Then, taking advantage of our cell culture model, we have sought to explore the impact of chronic exposure to cadmium (inhibitor of MMR activity), hydrogen peroxide (oxidizing agent) and several antioxidants on the dynamics of repeat expansions in somatic cells.

## Materials and Methods

### Mouse Kidney Cell Culture, Chronic Metal Ion Exposure and Antioxidant Treatment

The D2763Kc2 cell line is a clonal cell line derived from the kidney of a 6-month-old *Dmt*-D mouse, which recreates high levels of expansion-biased trinucleotide repeat instability *in vitro* (Gomes-Pereira et al., 2001). Cell cultures were maintained and passaged as previously described (Gomes-Pereira et al., 2001; Gomes-Pereira and Monckton, 2004). For metal ion treatment experiments a progenitor culture was split into multiple aliquots: six replicate no-metal ion controls, and six replicate cultures for each one of the compounds tested in this study (CdCl_2_, CoCl_2_, MnCl_2_, and ZnSO_4_). Similarly, six replicate cultures were continuously exposed to each individual antioxidant. All cultures were maintained in parallel throughout the course of the experiment. Control cultures were supplied with fresh medium every 2 or 3 days and cells were passaged when confluent at a 1:40 dilution, approximately weekly. For treated cultures, each metal compound and antioxidant was dissolved in complete growth medium and supplied to the cells. Treated cultures were supplied with fresh drug-supplemented medium every 2 to 3 days and the cells were passaged just as the no-treatment controls. Control and treated cells were cultured for a maximum of 73 days. Sublethal doses were selected in previous survival assays, using increasing concentrations of metal compounds and antioxidants.

### SP-PCR Amplification of Transgenic Trinucleotide Repeats

The degree of repeat length variation in each sample was assessed by sensitive small-pool PCR (SP-PCR) analysis, performed as previously described, using oligonucleotide primers DM-C and DM-BR (Monckton et al., 1995; Gomes-Pereira et al., 2004a). Each lane on the representative autoradiographs shown in the figures contains the amplification products of a single SP-PCR with ~5 to 20 genomic equivalents of input DNA. Each autoradiograph panel for an individual replicate culture is a cropped image derived from a separate autoradiograph and multiple images have been juxtaposed to aid comparison. The degree of repeat length variation observed in treated and control cell replicates at the time points indicated was schematically represented by box plots. The top and bottom of the boxes correspond to the third (Q3) and first quartiles (Q1), respectively and the line across the box displays the median. The lines extending from the top and the bottom of the boxes, include values that fall inside the lower and upper limits: Q1-1.5(Q3-Q1) and Q3+1.5(Q3-Q1), respectively. The median repeat length gain was determined by measuring the degree of variation in individual cultures and comparing this with the degree of variation measured in the progenitor culture. Expansion rates (in units of repeats gained per day) were calculated by dividing the median repeat gain over the number of days in culture, as previously described (Gomes-Pereira and Monckton, 2004).

### Quantification and Statistical Analysis of Repeat Size Variability

Single molecule SP-PCR analysis of 20 to 80 molecules per culture was used to determine the median repeat length in individual cultures. The median rates of expansion for each individual culture were calculated relative to the measured median repeat length in the progenitor culture at time zero, as described before (Gomes-Pereira and Monckton, 2004). Median expansion rates for each culture were calculated in terms of repeat number change per day. To compare the median rates of expansion between treated and control cultures we used the non-parametric Kruskal-Wallis for multiple comparisons, in an adaptation of the methods previously described (Gomes-Pereira and Monckton, 2004). Statistical analyses were performed with Prism 8 (GraphPad Software).

### Protein Sample Preparation and Western Blotting

Protein was extracted from cultured cells using EBC lysis buffer (50 mM Tris-HCl, 120 mM NaCl, 0.5% (v/v) NP-40) containing protease inhibitors (protease inhibitor cocktail for mammalian tissues, Sigma, cat. no. P8340). Aliquots of 50 to 100 μg of whole cell protein lysates were resolved by electrophoresis through a NuPAGE 4 to 12% Bis-Tris Gel (Invitrogen, cat. no. NP0321) and electroblotted onto Millipore Immobilon-P membranes (Millipore, cat. no. IPVH00010) at 30 V for 2 h in an XCellII Blot Module (Novex, cat. no. EI9051) in NuPAGE Transfer Buffer (Invitrogen, cat. no. NP0006-1). The membranes were blocked overnight at 4°C in 2.5% (w/v) or 5% (w/v) dried milk in TBST (20 mM Tris-HCl pH 7.6, 137 mM NaCl, 0.06% (v/v) Tween-20), then incubated for 2 h at room temperature in primary antibody. The membranes were washed four times for 15 min each in TBST, incubated for 1 h in secondary antibody at room temperature, and washed four times for 15 min each in TBST at room temperature. Antibody binding was visualized using SuperSignal West Pico Chemiluminescent Substrate (Pierce, cat. no. 34080). PCNA was detected using 400 ng ml^−1^ rabbit anti-PCNA polyclonal antibody (Santa Cruz Biotechnology, Cat# sc-7907, RRID: AB_2160375) in 5% (w/v) dried milk in TBST. MSH2 was detected using 100 ng ml^−1^ mouse anti-MSH2 monoclonal antibody (Millipore, Cat# NA27, RRID: AB_2266524) in 5% (w/v) dried milk in TBST. MSH3 was detected using 200 ng ml^−1^ goat anti-MSH3 polyclonal antibody (Santa Cruz Biotechnology, Cat# sc-5690, RRID: AB_2145131) in 5% (w/v) dried milk in TBST. MSH6 was detected using 250 ng ml^−1^ mouse anti-MSH6 monoclonal antibody (BD Biosciences, Cat# 610919, RRID: AB_398234) in 5% (w/v) dried milk in TBST. PMS2 was detected using 1 μg ml^−1^ mouse anti-PMS2 monoclonal antibody (BD Biosciences, Cat# 556415, RRID: AB_396410) in 5% (w/v) dried milk in TBST. MLH1 was detected using 2.5 μg ml^−1^ mouse anti-MLH1 monoclonal antibody (BD Biosciences, Cat# 554073, RRID: AB_395227) in 5% (w/v) dried milk in TBST. β-Tubulin was detected using 200 ng ml^−1^ rabbit anti-β-tubulin polyclonal antibody (Santa Cruz Biotechnology, Cat# sc-9104, RRID: AB_2241191). All proteins were detected using 20 ng ml^−1^ goat anti-rabbit (Santa Cruz Biotechnology, Cat# sc-2004, RRID: AB_631746), 8 ng ml^−1^ goat anti-mouse (Jackson ImmunoResearch Labs, Cat# 115-035-003, RRID: AB_10015289) or 4 ng ml^−1^ donkey anti-goat (Santa Cruz Biotechnology, Cat# sc-2020, RRID: AB_631728) horseradish peroxidase-conjugated secondary antibodies diluted in 2.5% (w/v) or 5% (w/v) dried milk in TBST. Incubation with Restore Western Blot Stripping Buffer (Pierce, cat. no. 21059) for 20 min at 40°C was performed to break antibody-antigen interactions and to subsequently re-probe the same membrane. Densitometric analysis of protein expression levels was performed using Kodak Digital Science 1D software using exposures in the linear range of signal intensity.

## Results

### Different Levels of Repeat Instability in Cultured Cells Are Associated With Variability in MMR Protein Expression

**Figure 1:**
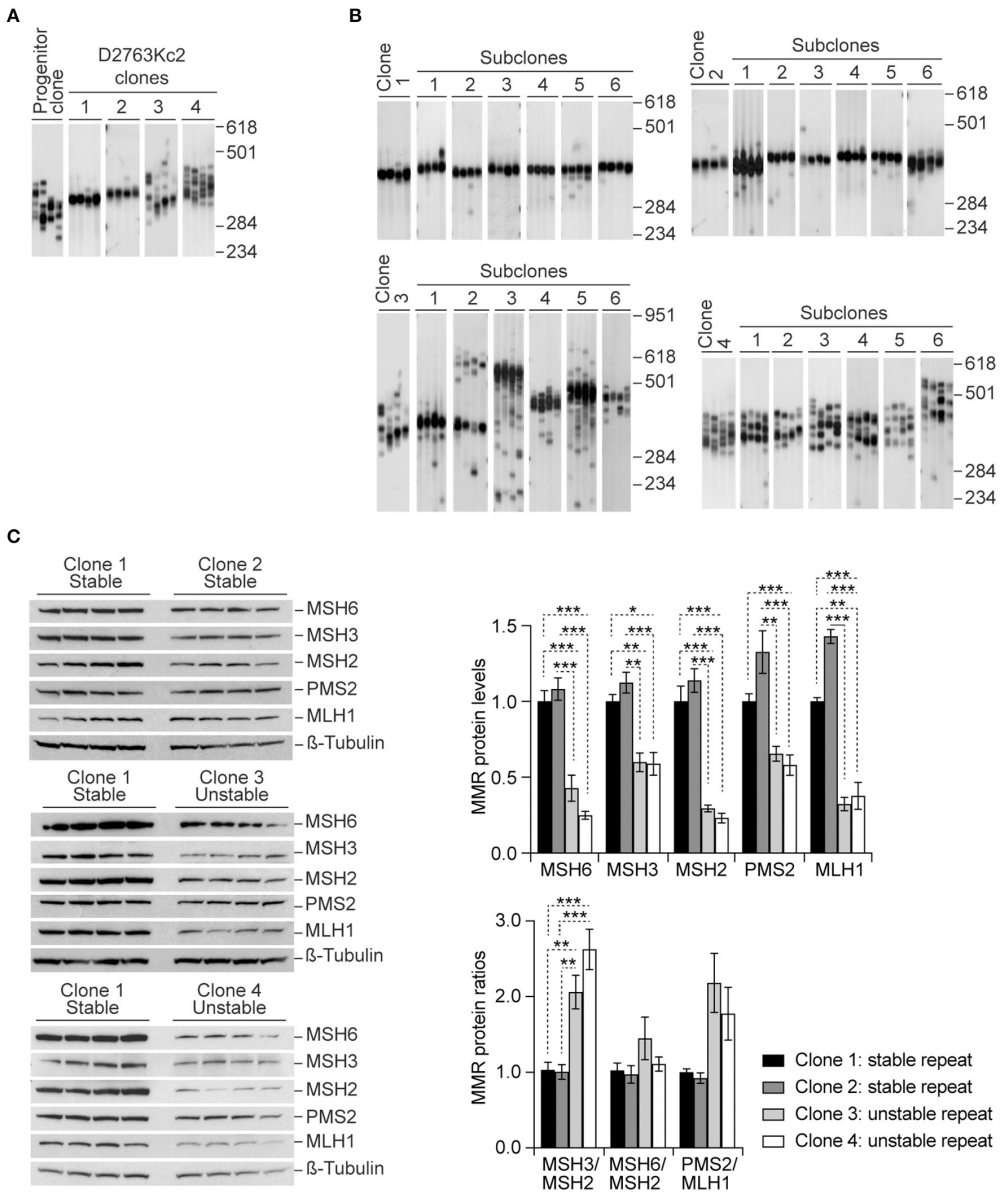
Higher CAG•CTG repeat size variability is associated with higher MSH3/MSH2 protein ratios in homogenous single cell clones. **(A)** Representative SP-PCR analyses of the expanded CAG•CTG trinucleotide repeat in D2763Kc2 cell clones. Single-cell clones were derived from the progenitor culture by limiting dilution and grown for 20 population doublings (~20 to 40 days). **(B)** Representative SP-PCRs of the expanded CAG•CTG repeat in sub-clones derived from four selected D2763Kc2 clones showing different repeat dynamics. For clarity, only six representative sub-clonal cultures are shown. The scale on the right indicates the DNA molecular weight markers converted into number of CAG•CTG repeats. **(C)** Quantification of MMR protein levels in selected D2763Kc2 clones. Independent single cell clones were established from the same progenitor culture. Four clones were selected for analysis. Four protein lysates were collected from each clone, electroblotted and probed with anti-MMR proteins. ß-Tubulin was used as loading control. The top graph shows the average protein levels relative to normalized control clone 1. The bottom graph shows the average MMR protein ratios relative to normalized control clone 1. The error bars indicate ± SEM. Statistically significant changes are indicated (*p < 0.05, **p < 0.01, ***p < 0.0015; Two-way ANOVA, Tukey *post-hoc* correction for multiple comparisons).

Different levels of somatic instability between tissues have been correlated with variability in the expression of MMR and BER proteins (Goula et al., 2009; Mason et al., 2014). However, trinucleotide repeat size dynamics is governed by a complex interplay between *cis* and *trans*-acting factors. It is therefore difficult to directly attribute the variability in somatic mosaicism between tissues to changes in the expression levels of DNA repair proteins alone. To further elucidate the correlation between MMR protein levels and repeat dynamics we derived single cell clones from the progenitor D2763Kc2 culture (Gomes-Pereira et al., 2001; Gomes-Pereira and Monckton, 2004) by limiting dilution, and identified four subclones that exhibited different degrees of somatic mosaicism, in spite of the similar proliferative capacity, as revealed by their population doubling time (PDT): while the CAG•CTG repeat tract was relatively stable in clones 1 and 2, it exhibited more pronounced repeat size variability in clones 3 and 4 (Figure 1A, Table 1). Importantly, repeat instability remained consistently low or high following further subcloning, suggesting intrinsic differences in DNA metabolism between the four clones selected (Figure 1B). Variability between subclones may result from the accumulation of spontaneous mutations and/or epigenetic modifications in cultured cells (Wilson and Jones, 1983; Giraldo et al., 2007). It was our hypothesis that some of these changes alter the MMR expression levels, affect DNA metabolism and modify the dynamics of trinucleotide repeats.

**Table 1:** Population doubling times of D2763Kc2 progenitor culture, and clones derived thereof.

**Clone/Subclone**	**PDT (h)**
D2763Kc2	23.9
Subclone 1	25.1
Subclone 2	27.0
Subclone 3	26.9
Subclone 4	26.6

Consistent with a driving role of MMR proteins in triplet repeat metabolism, western blot analysis revealed significantly different steady-state levels MMR protein between clones carrying repeats that were either stable (clones 1 and 2) or unstable (clones 3 and 4) (Figure 1C, Supplementary Figure 1, *p* < *0.001, Three-way ANOVA*). Surprisingly, comparison of individual protein levels revealed that the “unstable clones” 3 and 4 expressed significantly lower levels of MSH2, MSH3 and MSH6, when compared to the “stable clones” 1 and 2 (Table 2). In contrast, the levels of MSH2, MSH3 and MSH6 did not differ between “stable clones” 1 and 2, neither did they differ between “unstable clones” 3 and 4. We also found intriguing differences in the levels of PMS2 and MLH1 between the two classes of clones: PMS2 and MLH1 expression was also significantly lower in clones showing extensive somatic mosaicism. However, the ratios of MMR proteins were significantly different between “unstable” and “stable” clones. In particular, high levels of somatic mosaicism were associated with significantly higher MSH3/MSH2 protein ratios in “unstable” clones (3 and 4), relative to the “stable” clones (1 and 2). PMS2/MLH1 protein ratios were also higher in “unstable” clones, but the difference did not reach statistical significance (Figure 1C; Table 3).

**Table 2:** Statistical comparison of MSH6, MSH3, MSH2, PMS2 and MLH1 protein levels between clones.

		**Clone 1 Stable**	**Clone 2 Stable**	**Clone 3 Unstable**
**Clone 2 Stable**	MSH6	n.s. *p* = >0.9999		
	MSH3	n.s. *p* = 0.9990		
	MSH2	n.s. *p* = 0.9960		
	PMS2	n.s. *p* = 0.1248		
	MLH1	^**^p = 0.0063		
**Clone 3 Unstable**	MSH6	^***^p < 0.0001	^***^p < 0.0001	
	MSH3	^**^p = 0.00150	^**^p = 0.0002	
	MSH2	^***^p < 0.0001	^***^p < 0.0001	
	PMS2	n.s. *p* = 0.0765	^***^p < 0.0001	
	MLH1	^***^p < 0.0001	^***^p < 0.0001	
**Clone 4 Unstable**	MSH6	^***^p < 0.0001	^***^p < 0.0001	n.s. *p* = 0.9422
	MSH3	^*^p = 0.0104	^***^p < 0.0001	n.s. *p* > 0.9999
	MSH2	^***^p < 0.0001	^***^p < 0.0001	n.s. *p* > 0.9999
	PMS2	^**^p = 0.0080	^***^p < 0.0001	n.s *p* > 0.9999
	MLH1	^***^p < 0.0001	^***^p < 0.0001	n.s. *p* > 0.9999

* p < 0,05;

** p < 0.01;

*** p < 0.001;

*n.s., non-significant; Two-way ANOVA, Tukey's multiple comparison test*.

**Table 3:** Statistical comparison of MMR protein ratios between clones.

		**Clone 1 Stable**	**Clone 2 Stable**	**Clone 3 Unstable**
**Clone 2 Stable**	MSH3/MSH2	n.s. *p* = 0.9974		
	MSH6/MSH2	n.s. *p* = 0.9839		
	MSH3/MSH6	n.s. *p* = 0.9089		
	PMS2/MLH1	n.s. *p* = 0.7809		
**Clone 3 Unstable**	MSH3/MSH2	^*^p = 0.0413	^*^p = 0.0387	
	MSH6/MSH2	n.s. *p* = 0.5546	n.s. *p* = 0.4867	
	MSH3/MSH6	n.s. *p* = 0.5729	n.s. *p* = 0.9141	
	PMS2/MLH1	n.s. *p* = 0.1565	n.s. *p* = 0.1333	
**Clone 4 Unstable**	MSH3/MSH2	^*^p = 0.0188	^*^p = 0.0184	n.s. *p* = 0.4337
	MSH6/MSH2	n.s. *p* = 0.9193	n.s. *p* = 0.7959	n.s. *p* = 0.6898
	MSH3/MSH6	n.s. *p* = 0.0835	n.s. *p* = 0.0881	n.s. *p* = 0.5415
	PMS2/MLH1	n.s. *p* = 0.2960	n.s. *p* = 0.2462	n.s. *p* = 0.8625

* p < 0,05;

*n.s., non-significant; Two-way ANOVA, Tukey's multiple comparison test*.

In summary, our cell model system supports the view that instability of CAG•CTG repeats is correlated with pathways of DNA repair. Relative MMR proteins levels, in particular, appear to be key determinants of repeat dynamics. Motivated by these results we tested whether repeat length mutational dynamics can be modified by the exposure to metal ions reported to interfere with MMR activity (Jin et al., 2003).

### Effects of Cadmium on the Dynamics of Trinucleotide Repeats in Cultured Cells

To test if cadmium exposure was capable of modifying trinucleotide repeat dynamics, we treated D2763Kc2 mouse kidney cells to cadmium, as well as control divalent metal ions (cobalt, manganese and zinc). We carefully selected sublethal metal concentrations added to the cell culture medium in survival assays (Supplementary Figure 2): 2.0 μM was the highest concentration of CdCl_2_, CoCl_2_ and MnCl_2_ that did not affect the overall survival and PDT of D2763Kc2 cell cultures chronically exposed for 7 days (Table 4). Although the survival and PDT remained unaltered in the presence of 10 μM of ZnSO_4_, we used a zinc concentration of 2.0 μM in our experiments, identical to the other metals. The similar PDT between treated and control cultures excluded altered cell division rates as a contributing factor to any changes in trinucleotide repeat dynamics.

**Table 4:** Metal and antioxidant exposure and the dynamics of expanded CAG•CTG repeats.

**Treatment [drug concentration]**	**Time (days)**	**PD^a^**	**PDT^b^**	**Change in PDT (relative to controls)**	**Median expansion rate (repeats/day)**	**Change in median expansion rate (relative to controls)**	**Significance, *p*^d^**
Control	73	73	23.9	-	1.00	NA^c^	NA^c^
Manganese [2 μM]	73	73	23.9	0%	0.81	−19%	0.026
Cobalt [2 μM]	73	73	23.9	0%	1.01	+1%	0.974
Zinc [2 μM]	73	73	23.9	0%	1.18	+18%	0.158
Cadmium [2 μM]	73	73	23.9	0%	1.13	+13%	0.168
H_2_O_2_ [20 μM]	73	73	23.9	0%	0.92	−8%	0.612
Ethanol [0.1%]	73	73	23.9	0%	1.07	+7%	0.774
Melatonin [20 μM in 0.1% ethanol]	73	73	23.9	0%^e^	0.98	−8%^e^	0.528^e^
Ascorbic Acid [200 μM]	73	68	25.8	+8%	1.10	+10%	0.603
Trolox C [500 μM in 0.1% ethanol]	73	73	23.9	0%^e^	1.07	0%^e^	0.511^e^

a *PD, population doublings*.

b *PDT, population doubling time*.

c *NA, not applicable*.

d *Two-tailed Kruskal-Wallis test*.

e *Relative to ethanol vehicle controls*.

**Figure 2:**
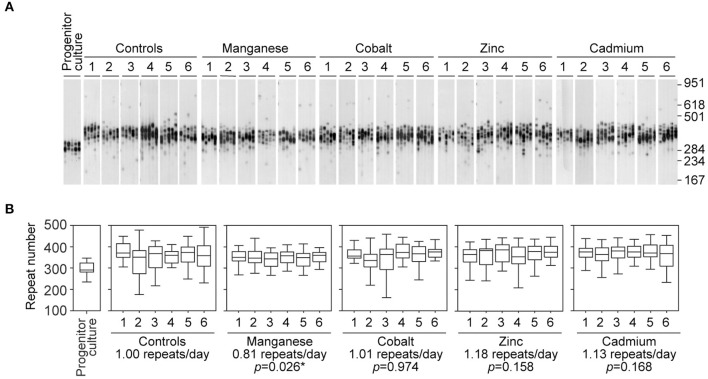
Chronic exposure to cadmium and other divalent metals does not greatly affect the dynamics of expanded CAG•CTG repeats. **(A)** The autoradiographs show representative SP-PCR analyses of expanded CAG•CTG repeats. Replicate D2763Kc2 cell cultures (1 to 6) were exposed to 2 μM of the metal compounds over a period of 73 days, corresponding to 73 population doublings. The progenitor culture from which all cells were derived at day zero is shown. The scale on the right displays the molecular weight markers converted into CTG repeat numbers. **(B)** The boxplots represent the degree of repeat length variability in treated and control cells following chronic exposure. Differences between the median expansion rates of control and treated cell cultures were analyzed and the *p*-values presented (Kruskal-Wallis test for multiple comparisons; *p < 0.05). A significant reduction in the repeat expansion rate was detected in the presence of manganese.

The original progenitor culture was split at time point zero to generate control and treated cell cultures. For each metal treatment six replicate cultures were established and maintained in parallel with six untreated controls. Control and treated D2763Kc2 cell cultures were then maintained for a period of 73 days, corresponding to 73 population doublings (PDT = 23.9 h). Following chronic exposure, the repeat length variability of each chemically treated culture was assessed by sensitive SP-PCR techniques and compared to the progenitor and control cultures grown in the absence of the metal (Figure 2). The transgenic repeat length continued to expand rapidly in control cells, with a median expansion rate of 1.0 repeat per day. Metal-treated cultures displayed median expansion rates varying from 0.81 to 1.18 repeats per day. We determined if the median expansion rates measured in control and treated cultures were statistically different, using single molecule analyses as previously described (Gomes-Pereira and Monckton, 2004; Gomes-Pereira et al., 2014). Cadmium exposure did not change the expansion rate of the trinucleotide repeat sequence, which continued to expand at a median expansion rate of 1.13 repeats per day that did not differ significantly from control cells (*p* = 0.168, Kuskal-Wallis test). Surprisingly, manganese-treated cells displayed a median repeat expansion of 0.81 repeats per day, representing a significant reduction of ~ 20% relative to untreated cells (*p* = 0.026, Kuskal-Wallis test), ~28% relative to cadmium-treated cultures (*p* = 0.0023, Kuskal-Wallis test) and ~ 31% relative to zinc-treated cultures (*p* = 0.0021, Kuskal-Wallis test).

It is conceivable than rather than affecting the median repeat length in culture, the exposure to cadmium changes the spread of repeat sizes carried by individual cells. We used the variance of repeat sizes in each replicate culture as a measure of repeat size dispersion, and tested if the chronic exposure to metals caused significant changes between treatments. In addition to the reduction in the expansion rate, treatment with manganese also decreased repeat size heterogeneity relative to controls (2226.0 ± 301.0, manganese; 4058.9 ± 26.4, control; *p* = 0.030, Kuskal-Wallis test). In conclusion, while cadmium did not detectably modify the somatic instability of CAG•CTG repeats in D2763Kc2 mouse kidney cells, manganese appeared to slow down trinucleotide repeat expansion in culture.

### Effects of Antioxidants on the Dynamics of Trinucleotide Repeats in Cultured Cells

**Figure 3:**
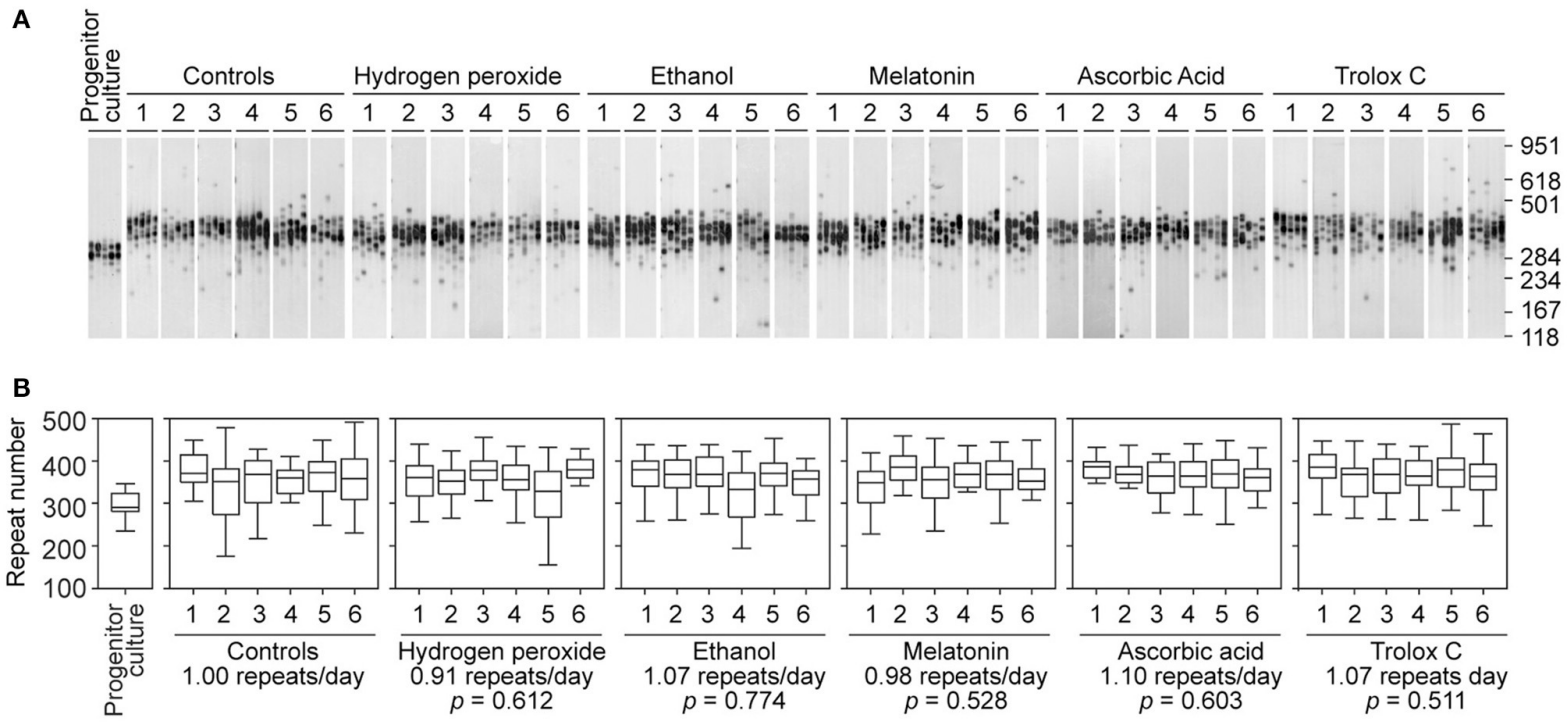
Continuous treatment with antioxidant molecules does not prevent repeat size expansions in a cell culture model system of unstable CAG•CTG repeats. **(A)** Representative SP-PCR analyses of the expanded CAG•CTG trinucleotide repeat in D2763Kc2 cells cultured for 73 population doublings (corresponding to 68 to 73 days) in the presence of hydrogen peroxide, ethanol (control solvent) or three different antioxidants. Six replicate D2763Kc2 cultures (1 to 6) were analyzed. The progenitor culture from which all cells were derived at day zero is also shown. The scale on the right displays the molecular weight markers converted into CTG repeat numbers. **(B)** The boxplots show the degree of repeat length variability in treated and control cells at the end of the treatment. None of the treatments tested resulted in a significant change of the repeat expansion rate. *p-*values are indicated (Kruskal-Wallis test for multiple comparisons).

To determine if antioxidants are capable of slowing down repeat expansion in our cell model of unstable trinucleotide sequences, we treated D2763Kc2 cells over 73 days with a panel of well-known primary antioxidants that exhibit efficient radical-scavenging activity: melatonin, ascorbic acid and Trolox C (an analog of vitamin E) (Simunkova et al., 2019). In parallel, long-term exposure to hydrogen peroxide was used to mimic conditions of chronic oxidative stress. As in previous experiments, we selected the highest dose of hydrogen peroxide and antioxidants that did not interfere with cell survival and PDT (Supplementary Figure 3, Table 4). The dynamics of expanded CAG•CTG repeats in D2763Kc2 cells was not affected by chronic exposure to hydrogen peroxide and antioxidants (Figure 3). Cells treated with antioxidants continued to expand at a rate of 0.91 to 1.10 repeats per day, which was not statistically different from the rate measured in untreated cells, or in cells exposed to ethanol (solvent control for melatonin and Trolox C). We then tested if repeat size variability (rather than repeat median) was different between treatments. Interestingly the variance of repeat sizes following ascorbic acid exposure was significantly lower relative to untreated control cells (2713.3 ± 129.7, ascorbic acid; 3390.7 ± 195.7, control; *p* = 0.0411, Kruskal-Wallis test). The other treatments did not change significantly the spread of repeat sizes. Together our data suggest that the dynamics of the expanded CAG•CTG sequence is not greatly altered by oxidizing agents or antioxidants in our cell culture model system of unstable triplet repeats.

## Discussion

Our main aim was to determine if the exposure to chemicals reported to reduce MMR activity and oxidative stress could modify the dynamics of expanded trinucleotide repeats. Our findings reveal that chronic exposure of our cell culture models of unstable CAG•CTG trinucleotides to sub-lethal doses of cadmium (a putative MMR inhibitor), as well as melatonin, ascorbic acid and Trolox C (well-known antioxidants) did not affect the somatic repeat expansion rate. We have however identified manganese, as a divalent metal that appears capable of stabilizing the repeat, and ascorbic acid as a water-soluble antioxidant that appears to reduce repeat size variability without affecting the overall net repeat expansion rate.

We first determined if the levels of somatic mosaicism correlated with MMR protein levels in cell culture. Cell clones displaying high degrees of repeat size variability did not express higher absolute levels of MMR proteins, but showed differences in the relative expression of MMR proteins: in particular, the clones exhibiting high levels of somatic mosaicism showed significantly higher MSH3/MSH2 protein ratios. The analysis of four individual clones produced results that are in agreement with the reported role of individual MMR proteins on the dynamics of CAG•CTG repeats. Ablation of MMR genes in different mouse models carrying unstable CAG•CTG sequences revealed that the deficiency of specific genes (*Msh2, Msh3, Mlh1, Mlh3*, and *Pms2*) can suppress somatic and/or germline instability (Manley et al., 1999; Van Den Broek et al., 2002; Gomes-Pereira et al., 2004b; Pinto et al., 2013; Schmidt and Pearson, 2016). These effects are gene-specific, since *Msh6* deficiency did not result in repeat stabilization and could even promote repeat expansion (Van Den Broek et al., 2002; Foiry et al., 2006; Dragileva et al., 2009). The opposing effects of *Msh3* and *Msh6* inactivation is consistent with the proposal that the dynamics of expanded trinucleotides could be dictated by the recognition and binding of two partially redundant MutS complexes: the MSH2•MSH3 complex promoting repeat expansion; and the MSH2•MSH6 complex mediating repeat deletion (Van Den Broek et al., 2002). Importantly, the stability of individual MSH3 and MSH6 proteins depends on their heterodimerization with MSH2. Hence, the low levels of MSH6 protein measured in “unstable” clones may provide more MSH2 to bind and stabilize MSH3, increasing the formation of expansion-promoting MSH2•MSH3 heterodimers.

We then sought to explore new pharmacological means to modify repeat dynamics. The chronic exposure of D2763Kc2 kidney cells to cadmium over long periods of time, at doses that appeared to inhibit MMR in human cell extracts by ~3–30% (Jin et al., 2003), did not detectably alter trinucleotide repeat dynamics, leaving expansion rates unchanged. We conclude that low, sub-lethal doses of cadmium do not perturb the processing of unstable trinucleotide repeats. However, we did not directly assess MMR activity in our treated cells and it is possible that, in contrast with the acute exposure protocols previously reported (Lützen et al., 2004), the chronic treatments described here may have triggered an adaptative response to cadmium exposure that obviated the inhibitory effect of acute exposure to cadmium on MMR. Alternatively, the lack of a stabilizing effect of cadmium might instead be due to the inability of this metal to target exclusively MMR components involved in the expansion mechanism. Cadmium is reported to inhibit the endonuclease activity of the MLH1•PMS2 complex (Sherrer et al., 2018) and PMS2 deficiency is associated with reduced CAG•CTG expansion rates (Gomes-Pereira et al., 2004b). However, unlike MLH3 (Pinto et al., 2013), PMS2 is not essential for expansion (Gomes-Pereira et al., 2004b). Thus, it is possible that any inhibitory effect on MLH1•PMS2 activity is compensated for by the MLH1•MLH3 complex, although given that PMS2 and MLH3 are highly related orthologs, MLH1•MLH3 may be similarly inhibited by cadmium. Cadmium also appears to non-specifically alter the binding and ATPase activity of the MSH2•MSH6 complex (Clark and Kunkel, 2004; Banerjee and Flores-Rozas, 2005; Wieland et al., 2009). However, the MSH2•MSH6 complex is not required for CAG•CTG repeat expansion (Van Den Broek et al., 2002) and it is possible that the MSH2•MSH3 heterodimer, that is required for expansion (Van Den Broek et al., 2002), is not affected by cadmium. Alternatively, through its broader non-specific impact on a variety of DNA repair enzymes (Wieland et al., 2009), cadmium may alter other aspects of the CAG•CTG expansion pathway and compensate for any direct effect on the MMR components of expansion.

Surprisingly, manganese appeared capable of reducing the expansion rate and repeat size variability of expanded CAG•CTG repeats, without changing cell population dynamics. Manganese promotes MLH1•PMS2 endonuclease activity (Kadyrov et al., 2006). Given the implication of MLH1 and PMS2 in the control of CAG•CTG repeat dynamics in mice (Gomes-Pereira et al., 2004b; Pinto et al., 2013) and in humans (Ciosi et al., 2019), it is likely that interfering with the activity of this MMR heterodimer will impact repeat dynamics. Alternatively, the effect of manganese on trinucleotide repeat dynamics might be mediated by impaired fidelity and *trans*-lesion DNA synthesis (Hays and Berdis, 2002; Vashishtha et al., 2016).

The chronic exposure of D2763Kc2 kidney cells to multiple antioxidants, at doses that do not interfere with global population dynamics, did not stabilize the expanded CAG•CTG repeat carried by these cells. Ascorbic acid however, appeared capable of reducing repeat size variability, lowering somatic mosaicism in culture. Trinucleotide repeat dynamics is the combined result of stepwise expansions and contractions of a limited number of repeat units (Higham et al., 2012). Therefore, it is possible to reduce repeat size variability independent of net expansion size, through changes in the relative frequency of expansion and contraction events, and/or changes in the magnitude of stepwise mutations.

Overall, our data do not support the use of antioxidant scavenging systems to suppress somatic expansion. In agreement with these results, we previously showed that chronic exposure of D2763Kc2 to hydrogen peroxide cells did not induce repeat expansions (Gomes-Pereira and Monckton, 2004). However, our observations contrast with findings in HD fibroblasts (Kovtun et al., 2004) and mouse embryonic stem cells (Jonson et al., 2013) where hydrogen peroxide treatment appeared to increase expansion rates. While we have treated multiple replicates continuously (73 days) with low doses of hydrogen peroxide (20 μM), in an attempt to mimic chronic environmental stress, others have carried out acute exposure (30 min) with higher doses (150 to 1,000 μM), leaving the cells to recover for days before repeat length assessment (Kovtun et al., 2004; Jonson et al., 2013). It is also unclear if these experiments were replicated to control for stochastic variation and/or clonal selection and cell-expansion events. Alternatively, in the absence of direct measures of oxidative damage in our experiments, we cannot exclude the possibility that chronic exposure to oxidants triggered an adaptative response to reduce DNA damage and minimize genotoxicity.

In summary, chronic exposure to cadmium and antioxidants did not stabilize an expanded trinucleotide repeat in our cell culture model system. We thus anticipate that more efficacious and specific inhibitors of the expansion pathway may need to be rationally designed to modify repeat dynamics safely and efficiently for therapeutic benefit.

## Data Availability Statement

The original contributions presented in the study are included in the article/Supplementary Material, further inquiries can be directed to the corresponding author/s.

## Author Contributions

MG-P: experimental work, data acquisition, and preparation of figures. MG-P and DGM: study design/interpretation, data analysis, and manuscript preparation. All authors contributed to the article and approved the submitted version.

## Conflict of Interest

DGM had been a scientific consultant and/or received an honoraria/stock options from AMO Pharma, Biogen Idec, Charles River, LoQus23, Small Molecule RNA, Triplet Therapeutics and Vertex Pharmaceuticals, and held research contracts with AMO Pharma and Vertex Pharmaceuticals. The remaining author declares that the research was conducted in the absence of any commercial or financial relationships that could be construed as a potential conflict of interest.

## Supplementary Material

Supplementary Figure 1 Western blot detection of MMR proteins in D2763Kc2 clones. Uncropped western blots used to prepare Figure 1. Four protein samples collected from each clone were electrophoresed, electroblotted and probed with anti-MMR proteins. Two antibodies were often combined to detect different proteins simultaneously. ß-Tubulin was used as a loading control.

Supplementary Figure 2 Cell survival in the presence of metal compounds. Semi-logarithmic representation of the mean cell survival (±SD) of D2763Kc2 duplicate cultures exposed to increasing concentrations of metal compounds, over a period of seven days. Concentrations were plotted using a logarithmic scale. Cell survival was assessed by a trypan blue exclusion assay.

Supplementary Figure 3 Cell survival in the presence of oxidants and antioxidants. Semi-logarithmic representation of the mean cell survival (±SD) of D2763Kc2 duplicate cultures exposed to increasing concentrations of hydrogen peroxide and multiple antioxidant compounds, over a period of seven days. Ethanol was used as control vehicle. Concentrations were plotted using a logarithmic scale. Cell survival was assessed by a trypan blue exclusion assay.
