# Supplementary figures and images for: Chronic Exposure to Cadmium and Antioxidants Does Not Affect the Dynamics of Expanded CAG•CTG Trinucleotide Repeats in a Mouse Cell Culture System of Unstable DNA
Source: Front Cell Neurosci. 2021 Feb 2;14:606331. doi: 10.3389/fncel.2020.606331 (PMC7884634; doi:10.3389/fncel.2020.606331)

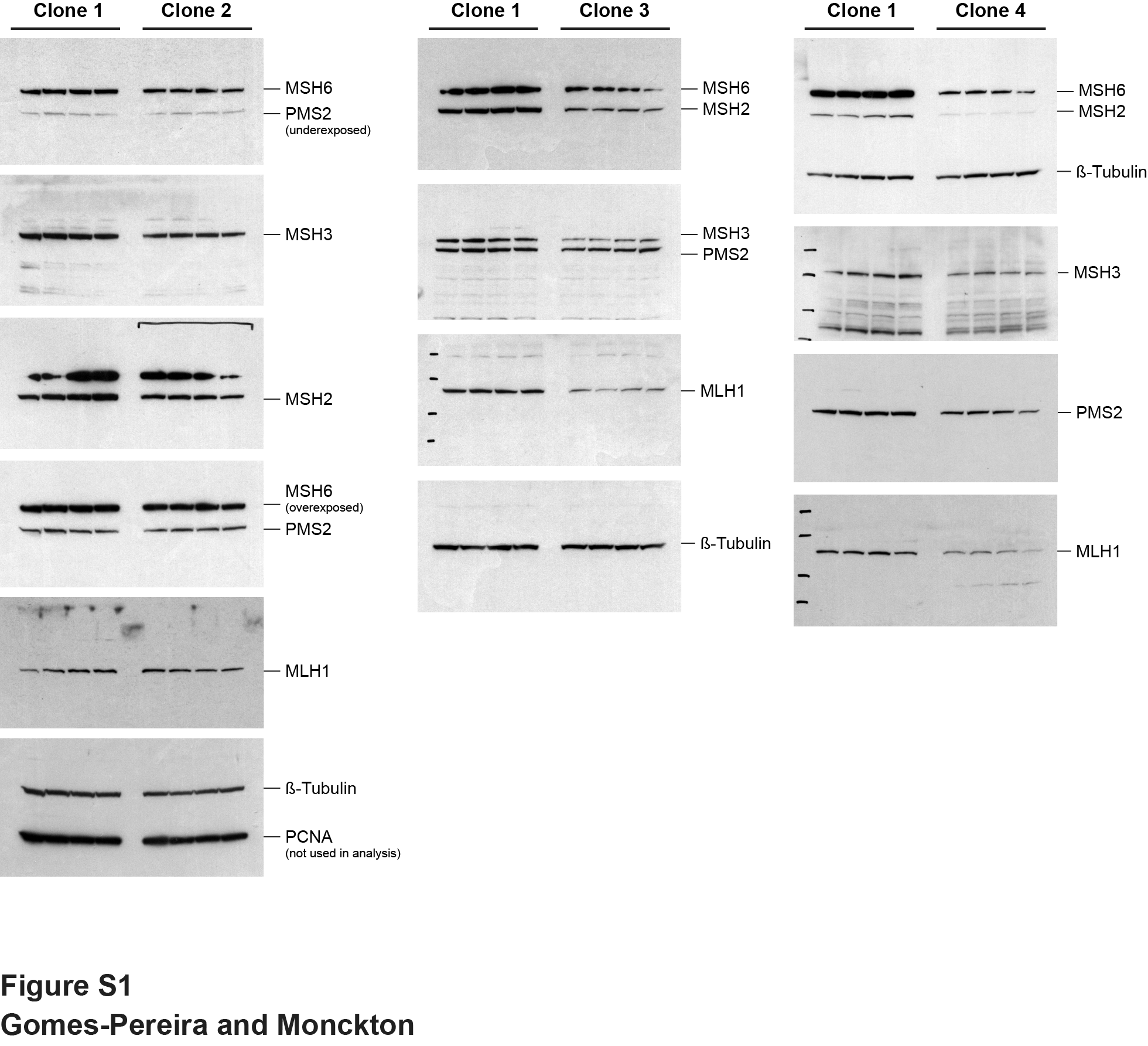

Supplement: Supplementary Figure 1 — Western blot detection of MMR proteins in D2763Kc2 clones. Uncropped western blots used to prepare Figure 1. Four protein samples collected from each clone were electrophoresed, electroblotted and probed with anti-MMR proteins. Two antibodies were often combined to detect different proteins simultaneously. ß-Tubulin was used as a loading control. [file Image_1.TIF]

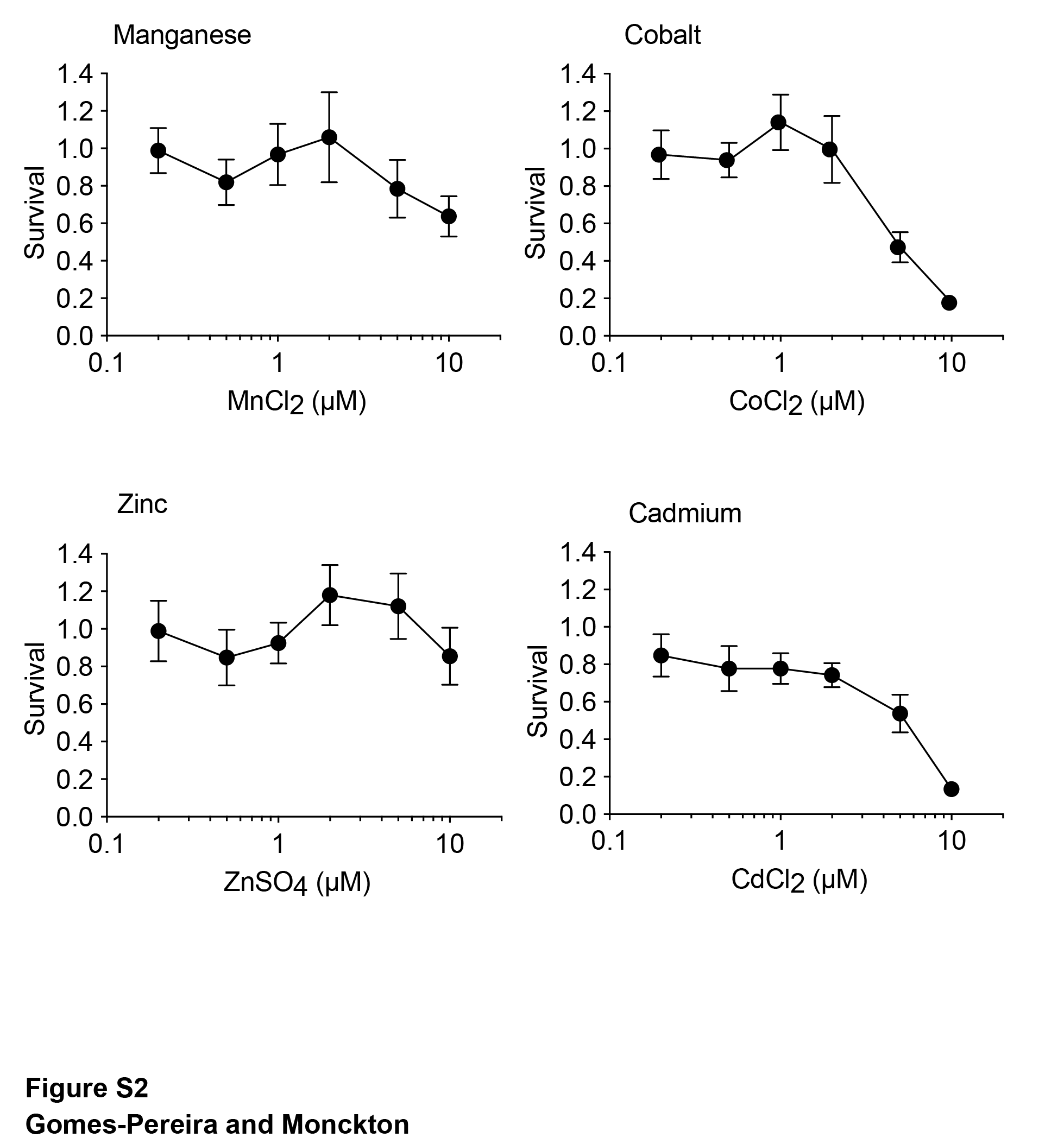

Supplement: Supplementary Figure 2 — Cell survival in the presence of metal compounds. Semi-logarithmic representation of the mean cell survival (±SD) of D2763Kc2 duplicate cultures exposed to increasing concentrations of metal compounds, over a period of seven days. Concentrations were plotted using a logarithmic scale. Cell survival was assessed by a trypan blue exclusion assay. [file Image_2.TIF]

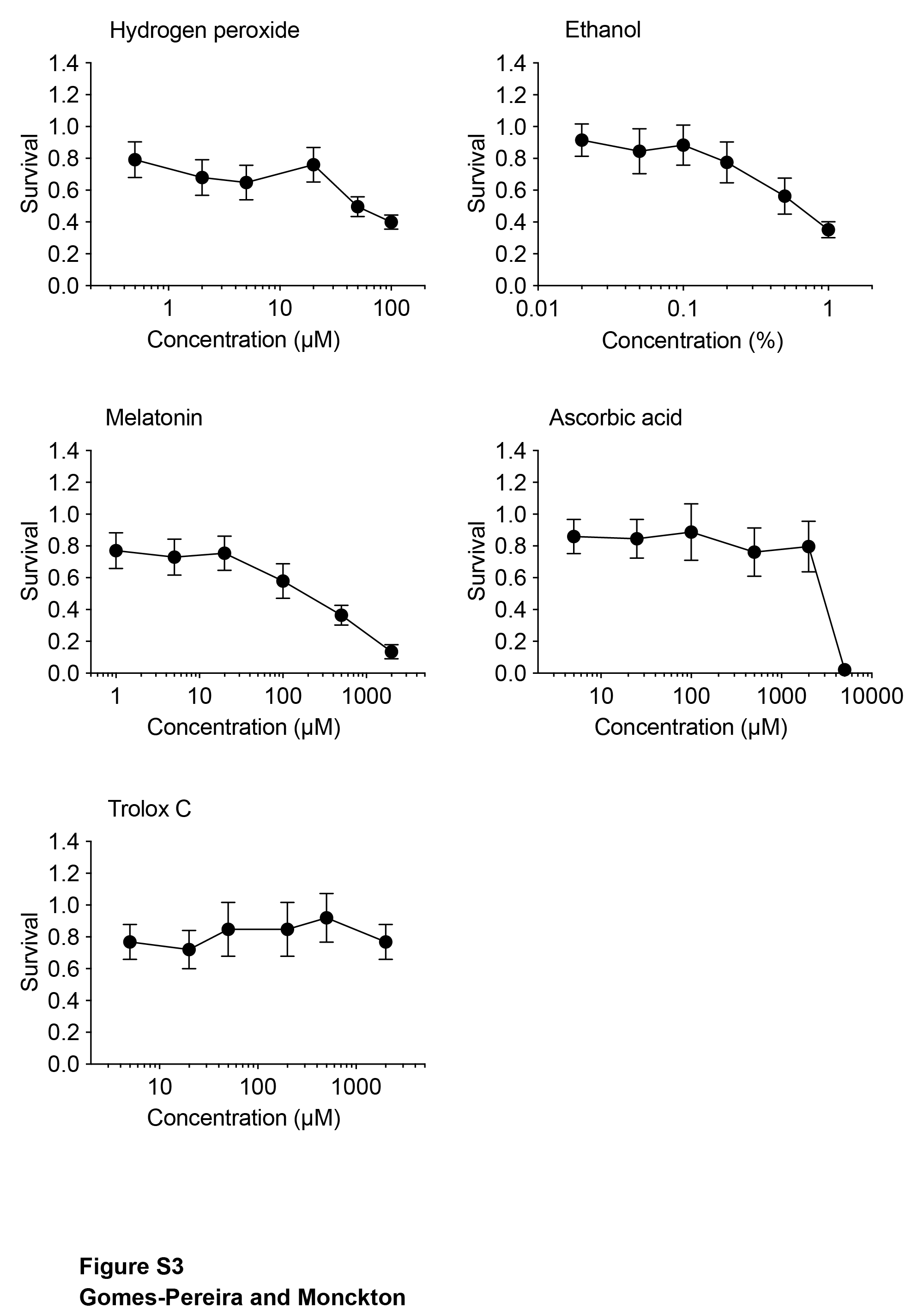

Supplement: Supplementary Figure 3 — Cell survival in the presence of oxidants and antioxidants. Semi-logarithmic representation of the mean cell survival (±SD) of D2763Kc2 duplicate cultures exposed to increasing concentrations of hydrogen peroxide and multiple antioxidant compounds, over a period of seven days. Ethanol was used as control vehicle. Concentrations were plotted using a logarithmic scale. Cell survival was assessed by a trypan blue exclusion assay. [file Image_3.TIF]
